# Effects of Gestational Diabetes Mellitus on Maternal and Neonatal Outcomes: A Prospective Cohort Study in Northern Sri Lanka

**DOI:** 10.7759/cureus.91727

**Published:** 2025-09-06

**Authors:** Kandiah Guruparan, Piraveena Raghavan, Yalini Guruparan

**Affiliations:** 1 Department of Obstetrics and Gynaecology, Faculty of Medicine, University of Jaffna, Jaffna, LKA; 2 Department of Pharmacology, Faculty of Medicine, University of Jaffna, Jaffna, LKA

**Keywords:** blood glucose control, fetal complications, gestational diabetes mellitus, maternal complications, neonatal complications

## Abstract

Introduction: Gestational diabetes mellitus (GDM) has emerged as a significant global public health concern due to its association with adverse maternal and neonatal outcomes. The aim of the study was to assess the effects of GDM on maternal and neonatal outcomes.

Methods: A prospective cohort study was conducted among 216 pregnant women diagnosed with GDM who were receiving care at Teaching Hospital Jaffna. Women with pre-existing diabetes were excluded from the study. Participants were followed from the time of GDM diagnosis until six weeks postpartum. Data were collected using structured questionnaires and a review of clinical records. Descriptive statistics were used to summarize the demographic and clinical characteristics. The chi-square test was used to assess the association between blood glucose control and adverse maternal and neonatal outcomes. A p-value ≤0.05 was considered statistically significant.

Results: The majority of participants (61.1%; n = 132) were aged 35 years or older. More than two-thirds of the participants (67.6%; n=146) were obese. The majority were multiparous (62%; n = 134), and GDM was diagnosed during the second trimester of pregnancy (61.1%; n = 132). A total of 17 mothers (7.9%) experienced a miscarriage during the current pregnancy. Maternal and neonatal complications were reported in 60 (27.8%) and 29 (14.6%) cases, respectively. A significant association was found between blood glucose control and both maternal (p < 0.001) and neonatal complications (p = 0.007).

Conclusion: Despite the provision of multidisciplinary antenatal care for diabetes, women with GDM remain at increased risk for maternal and neonatal complications. Greater emphasis is required on targeting modifiable risk factors, particularly by enhancing adherence to diabetes management strategies during pregnancy.

## Introduction

Gestational diabetes mellitus (GDM) is defined as “any degree of glucose intolerance with onset or first recognition during pregnancy” [1], and it remains one of the most common medical complications during pregnancy [2]. Gestational diabetes mellitus affects a substantial proportion of pregnant women worldwide, with prevalence estimates ranging from 1% to 14% [3]. According to available data, the prevalence of GDM in Sri Lanka was 13.9% [4].

Women with GDM are at an eightfold increased risk of experiencing adverse outcomes during pregnancy and delivery [5]. They are more likely to develop pregnancy-induced hypertension (PIH), undergo cesarean delivery, and experience complications such as vaginal candidiasis, urinary tract infections, and prelabor rupture of membranes, as well as antepartum and postpartum hemorrhage [6]. Neonates born to women with GDM are at increased risk of complications such as miscarriage, macrosomia, respiratory distress, neonatal jaundice, and admission to neonatal care units [6,7]. Early detection of GDM and good glycemic control were shown to decrease the incidence of diabetes-related complications [8]. Therefore, this study aimed to assess the effects of GDM on maternal and neonatal outcomes.

In Sri Lanka, all pregnant women are screened for gestational diabetes in the first trimester booking visit and late second trimester (24-28 weeks) routinely. [9] In high-risk clinical suspicion, screening for GDM is done in the third trimester as well.

## Materials and methods

Study setting and participants

A prospective cohort study was conducted among pregnant women who were recruited from the antenatal clinics of Teaching Hospital Jaffna. The study was conducted at antenatal clinics and obstetric wards at Teaching Hospital Jaffna, which is the largest tertiary care hospital in the Northern Province of Sri Lanka and offers primary, secondary, and tertiary health care services to the people residing in the Jaffna district. Participants who were aged 18 years and above with a singleton pregnancy were recruited at the time of diagnosis of GDM. They were followed up on every month until six weeks after delivery. Women who had pre-existing diabetes mellitus were excluded from this study.

Sample size and sampling

Sample size was calculated according to the Fernando et al. study [10]. The calculated sample size after adding 10% non-respondents was 216. Participants who were diagnosed with GDM were recruited consecutively till the required number (216) was reached.

Study instruments

A pre-tested structured questionnaire and data collection sheet were developed in English and translated into Tamil using the forward-backward translation method to ensure accuracy and cultural relevance. The questionnaire included sections on socio-demographic variables (age, level of education, occupational status, average monthly income), current pregnancy details (gravidity, parity, number of children), and previous pregnancy history (including whether the previous pregnancy was affected by GDM). It also collected information on delivery details of the current pregnancy, as well as any maternal complications (such as miscarriage, PIH, antepartum hemorrhage [APH], prelabor rupture of membranes [PROM], mode of delivery, and postpartum hemorrhage [PPH]) and neonatal complications (including respiratory distress syndrome, jaundice, infection, and hypoglycemia).

Study variables

Outcome Variables

Adverse maternal outcome was defined as the occurrence of one or more of the following: PIH, PROM, APH, and PPH. Pregnancy-induced hypertension is defined as systolic blood pressure ≥ 140 mmHg and/or diastolic blood pressure ≥ 90 mmHg after 20 weeks of pregnancy [11]. Miscarriage is defined as expulsion from its mother of an embryo or fetus weighing 500 g or less, corresponding to a gestational age of up to 20 completed weeks of gestation with no signs of life [12]. Prelabor rupture of membranes refers to rupture of membranes before the onset of labor (before 37 weeks of gestation) [13]. Antepartum hemorrhage is defined as bleeding from or into the genital tract, occurring from 24 weeks of pregnancy and prior to the onset of labor [14]. Postpartum hemorrhage is defined as a blood loss of 500 ml or more within 24 hours after birth [15].

Primary Variable

The diagnosis of GDM is made when one or more of the values of plasma glucose level are met (fasting: ≥ 100 mg/dl, 1 hour: ≥180mg/dl, 2 hours: ≥ 140 mg/dl, 1 or more positive values) following a 75 g oral glucose load [16].

Data collection

Data were collected between September 2023 and August 2024. Two pre-intern doctors collected the data. They were trained by the primary investigator in administering the questionnaire and recording the data to ensure the uniform application of the questionnaire. Pre-pregnancy weight was collected from the antenatal records.

Data analysis

Descriptive statistics were used to summarize the demographic and clinical characteristics of the study population. The chi-squared test was used to find out the association between blood glucose control and maternal and neonatal complications. A p-value of ≤0.05 was considered statistically significant.

Ethical considerations

Approval was obtained from the Ethics Review Committee, Faculty of Medicine, University of Jaffna, Sri Lanka [Ref. No. J/ERC/23/145/NDR/0294]. Administrative approvals were obtained from relevant authorities. Informed written consent was obtained from the participants prior to recruitment. Participants’ contact details were required for communication purposes and were collected and stored separately.

## Results

A total of 216 women diagnosed with GDM were recruited at antenatal clinics at the time of diagnosis. The mean age of the participants was 37.49 ± 6.97 years, ranging from 21 to 48 years. The majority (61.1%; n=132) were aged 35 years or older. More than half of the participants (55.6%; n=120) belonged to the low household income group, and around half (51.9%; n=112) had primary education. Socio-demographic characteristics of the participants are shown in Table 1.

**Table 1:** Socio-demographic characteristics of the participants

Variables	Number (%)
Age (years)	
18-25	16 (7.4)
26-34	68 (31.5)
≥35	132(61.1)
Monthly house hold income (Sri Lankan rupees)	
Low (≤50,000)	120 (55.6)
Middle (50,001 - 100,000)	50 (23.1)
Higher (> 100,000)	46 (21.3)
Highest educational level	
Primary	112 (51.9)
Secondary	80 (37)
Higher	24 (11.1)

More than two-thirds of the participants (67.6%; n=146) were obese. The majority of participants were multiparous (62%; n=134) and were diagnosed with GDM during the second trimester of pregnancy (61.1%; n=132). More than two-thirds of the mothers (72.7%; n=157) monitored their blood glucose levels at home, and around half (54.2%; n=117) achieved satisfactory glycemic control. More than one-third of them (44%; n=95) maintained good glycemic control with diet alone. Thirty-five mothers (16.2%) Thirty-five mothers (16.2%) had a history of GDM in a previous pregnancy, and around one-fifth of the mothers (18.5%) had a family history of diabetes (Table 2).

**Table 2:** Information related to pregnancy and gestational diabetes mellitus BMI: body mass index, GDM: gestational diabetes mellitus, POA: period of amenorrhea

Variables	Number (%)
Maternal pre-pregnancy BMI (kg/m^2^)	
Normal weight (18.5 -22.9)	36(16.7)
Overweight (23-24.9)	34 (15.7)
Obesity (≥ 25)	146 (67.6)
Parity	
Nulliparous	82 (38)
Multiparous	134 (62)
POA at the time of GDM diagnosis	
First trimester	32 (14.8)
Second trimester	132(61.1)
Third trimester	52 (24.1)
Blood glucose monitoring	
Home based	157 (72.7)
Hospital based	59 (27.3)
Blood glucose control	
Satisfactory	117 (54.2)
Unsatisfactory	99 (45.8)
Mode of GDM control	
Diet alone	95 (44)
Metformin	62 (28.7)
Insulin	25 (11.6)
Metformin and Insulin	34(15.7)
Past history of GDM	
Yes	35 (16.2)
No	181 (83.8)
Family history of GDM/DM	
Yes	41 (18.5)
No	175(81.5)

Seventeen mothers (7.9%) experienced a miscarriage during the current pregnancy. Around half of the mothers (49.2%, n=98) underwent cesarean section. Maternal complications were reported in 60 cases (27.8%). Among the 199 neonates, 29 (14.6%) experienced complications (Table 3).

**Table 3:** Information related to current pregnancy outcome and mode of delivery

Variables	Number (%)
Miscarriage	
Yes	17 (7.9)
No	199 (92.1)
Mode of delivery	
Normal vaginal delivery	80 (40.2)
Assisted vaginal delivery	21 (10.6)
Caesarean section	98 (49.2)
Maternal complications	
Yes	60 (27.8)
No	156 (72.2)
Neonatal complications	
Yes	29 (14.6)
No	170 (85.4)

**Figure 1:**
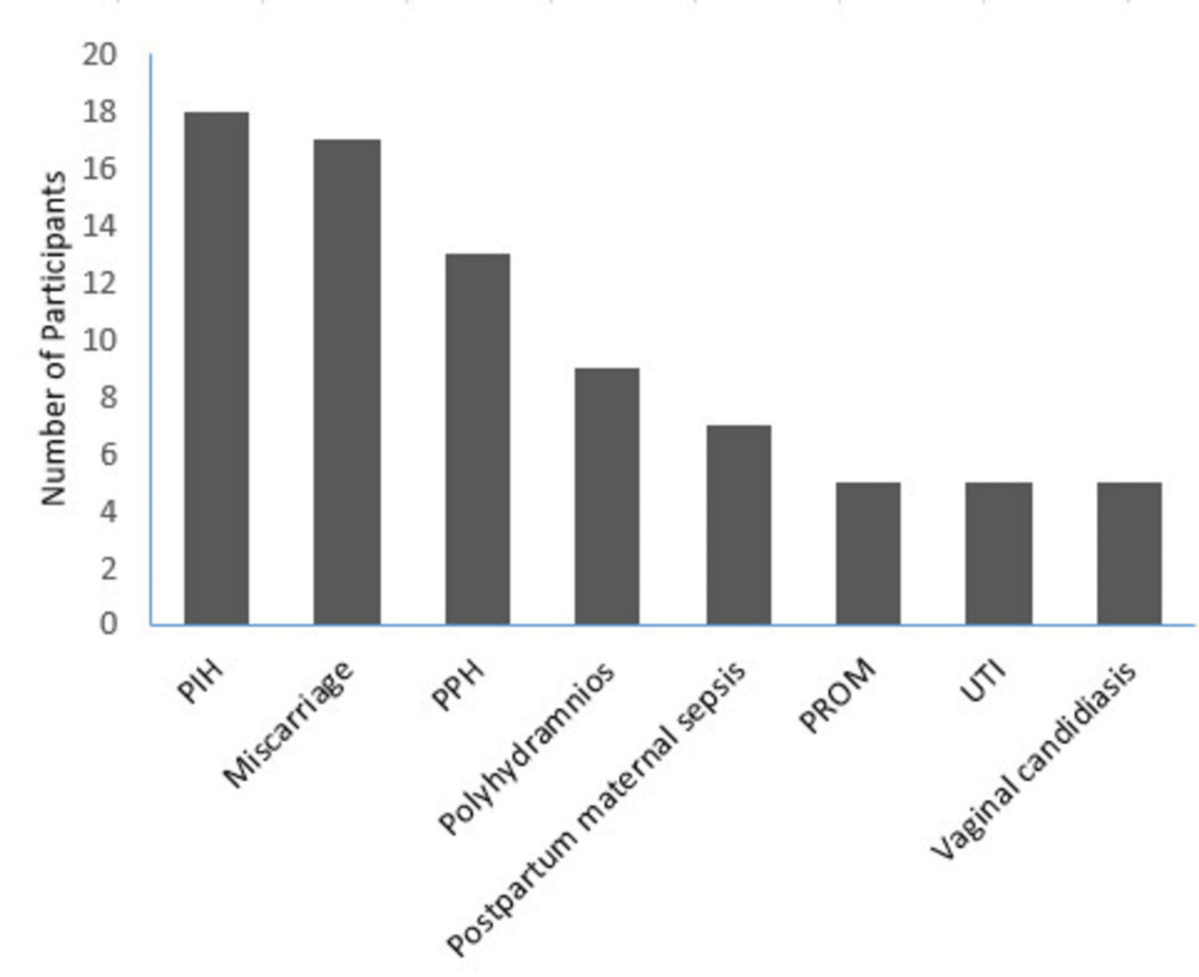
Maternal complications among mothers with gestational diabetes PIH: pregnancy-induced hypertension, PPH: postpartum hemorrhage, PROM: Prelabour rupture of membranes, UTI: Urinary tract infection

Pregnancy-induced hypertension and miscarriage were the commonly reported maternal complications. The distribution of maternal complications is depicted in Figure 1.

**Figure 2:**
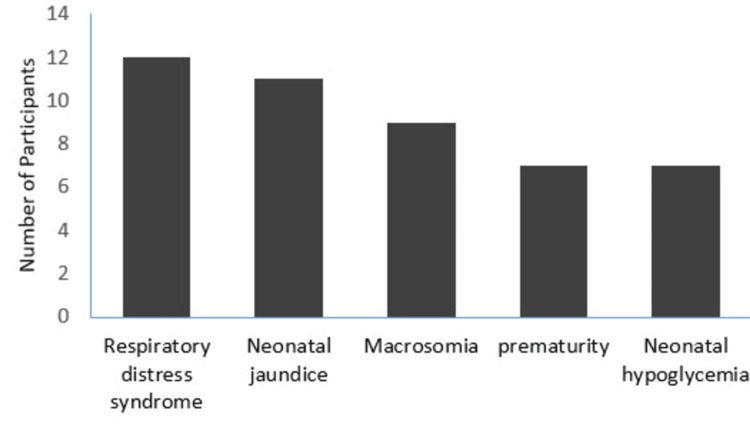
Neonatal complications among mothers with gestational diabetes

Respiratory distress syndrome and neonatal complications were commonly reported. Figure 2 illustrates the distribution of neonatal complications.

A significant association was found between blood sugar control and maternal complications (χ2= 19.54, p < 0.001), as well as fetal complications (χ2 = 7.22, p = 0.007) (Table 4).

**Table 4:** Control of blood glucose and maternal and fetal complications *A chi-square test of independence was done to find out the relation between blood glucose control and maternal complications. The chi-square statistic (χ2) is 19.54. The p-value is < 0.001. The result is statistically significant at p < 0.05. * A chi-square test of independence was done to find out the relation between blood sugar control and fetal complications. The chi-square statistic (χ2) is 7.22. The p-value is 0.007. The result is statistically significant at p < 0.05.

	Blood glucose control		Chi-square value	p-value
	Satisfactory	Unsatisfactory		
Maternal complication				
Yes	18	42	19.54	
No	99	57		
Fetal complication				
Yes	9	20	7.22	0.007
No	108	79		

## Discussion

This prospective study enrolled 216 pregnant women diagnosed with GDM, who were followed from diagnosis until delivery.

In this study, advanced maternal age (≥35 years) emerged as a notable predictor of GDM. Similar observations were reported in studies conducted in Sri Lanka [17], India [18], Pakistan [19], Qatar [20], Saudi Arabia [21], and Tanzania [22]. Studies from Malaysia [23] and Iran [24] revealed that women over the age of 30 years were at an increased risk of developing GDM compared to younger women. These observations strengthen the existing evidence of the association between advanced maternal age and GDM.

The present study identified that increasing maternal BMI (≥25 kg/m²) was associated with a higher risk of developing GDM. Comparable findings were reported in studies conducted in Sri Lanka [4,17], India [18], Bangladesh [25], Pakistan [19], Qatar [20], and Saudi Arabia [21]. These findings reinforce the established link between higher maternal BMI and the risk of developing GDM.

The current study revealed that individuals with a family history of diabetes mellitus were more likely to develop GDM. Similarly, studies conducted in Sri Lanka [17], Qatar [20], and Tanzania [22] also found a strong link between a family history of diabetes and the risk of developing GDM. This evidence further adds to the existing literature that supports the association between familial diabetes and increased risk of GDM.

In addition, the majority of the GDM cases in this study were diagnosed during the second trimester. A consistent pattern was observed in studies conducted in India [26], Pakistan [19], and Qatar [20], all indicating a higher incidence of GDM diagnoses during this period. These results further strengthen the existing literature, supporting the association between gestational age and the onset of GDM.

Glycemic control remains a key determinant in the prognosis of GDM. In our study, around half of the participants achieved satisfactory glycemic control during pregnancy. This nearly equal split highlights that a significant proportion of women remain at risk for adverse outcomes despite antenatal monitoring.

In this study, dietary intervention alone effectively controlled GDM in 44% of the participants. Comparable findings were found in studies conducted in India [27]. However, the Italian study paradoxically found that diet-only management was associated with a higher incidence of maternal complications. In the present study, outcomes were not analyzed based on treatment modality; however, poor glycemic control was generally associated with an increased risk of maternal complications. In contrast, a study from Bangladesh [25] reported that the majority of women with GDM were managed with insulin. This variation could be attributed to differences in patient characteristics or healthcare practices that influence the threshold for initiating pharmacological therapy.

In our study, 49.2% of women with GDM underwent cesarean section, while 40.2% had vaginal deliveries, reflecting a high rate of operative delivery. These findings closely align with a study conducted in India by Stuti Bahl et al. [28], which reported similar proportions, and with a study conducted in the United Kingdom by Karkia et al. [7], where cesarean section and vaginal deliveries were nearly equal (48.8% vs. 51.2%). Additionally, a study from Bangladesh by Shahnaz Akhter et al. [25] reported an even higher cesarean rate of 76% among women with GDM. In contrast to the above studies, a study conducted in Qatar by Bener et al. [20] reported a significantly lower cesarean section rate of 17.1% in this population. Collectively, these findings highlight both regional variations and the overall global trend of increased cesarean section rates in pregnancies complicated by GDM.

Our study revealed a substantial burden of maternal complications among women with GDM, affecting 27.8% of the cohort. The most observed complications were PIH (8.3%), miscarriage (7.9%), PPH (6%), and polyhydramnios (4.6%). A similar proportion of women with GDM experienced maternal complications in an Indian study; however, the types of complications differed. In that study, complications during labor (25%) and PIH (9%) were the most frequently reported [29]. A slightly higher proportion of maternal complications (36%) was reported in a Bangladeshi study, with pre-eclampsia (18%) and preterm labor (12%) being the most common [25]. The reason for these differences in the types and frequency of maternal complications could be attributed to variations in study populations, quality of antenatal care, and the timing and effectiveness of GDM management across different settings.

In this study, 14.6% of neonates born to mothers with GDM experienced complications. The most frequently observed neonatal complications were respiratory distress (6%), neonatal jaundice (5.5%), macrosomia (4.5%), and prematurity (3.5%). A greater proportion of adverse neonatal outcomes was noted in an Indian study; however, the types of complications remained similar. In that study, prematurity (11%) and respiratory distress (11%) were the most commonly documented conditions [29]. A Qatari study found a higher occurrence of such complications, with prematurity, neonatal jaundice, and birth trauma being the most frequently observed [20]. Differences in maternal profiles, clinical practices, and healthcare infrastructure may account for the observed variation in neonatal complication rates.

This study had several limitations. Being hospital-based and conducted in a single tertiary care setting, its findings may not be generalizable to the broader population. Additionally, the follow-up period was limited to six weeks postpartum, precluding long-term maternal or neonatal outcome assessment. Despite these limitations, the study offers meaningful insights into GDM-related challenges specific to the Northern Province of Sri Lanka and provides a baseline for further region-specific interventions.

## Conclusions

Although multidisciplinary antenatal diabetic care is provided, women with GDM continue to experience higher rates of maternal and neonatal complications. Enhanced focus is needed on addressing modifiable factors, particularly improving adherence to diabetes management plans among pregnant women.
